# Image Classification of Wheat Rust Based on Ensemble Learning

**DOI:** 10.3390/s22166047

**Published:** 2022-08-12

**Authors:** Qian Pan, Maofang Gao, Pingbo Wu, Jingwen Yan, Mohamed A. E. AbdelRahman

**Affiliations:** 1 Key Laboratory of Agricultural Remote Sensing, Ministry of Agriculture and Rural Affairs/Institute of Agricultural Resources and Regional Planning, Chinese Academy of Agricultural Sciences, Beijing 100081, China; 2 Key Laboratory of Digital Signal and Image Processing of Guangdong Province, Shantou University, Shantou 515063, China; 3 Division of Environmental Studies and Land Use, National Authority for Remote Sensing and Space Sciences (NARSS), Cairo 11769, Egypt

**Keywords:** wheat rust, ensemble learning, CNN, snapshot ensembling, SGDR-S

## Abstract

Rust is a common disease in wheat that significantly impacts its growth and yield. Stem rust and leaf rust of wheat are difficult to distinguish, and manual detection is time-consuming. With the aim of improving this situation, this study proposes a method for identifying wheat rust based on ensemble learning (WR-EL). The WR-EL method extracts and integrates multiple convolutional neural network (CNN) models, namely VGG, ResNet 101, ResNet 152, DenseNet 169, and DenseNet 201, based on bagging, snapshot ensembling, and the stochastic gradient descent with warm restarts (SGDR) algorithm. The identification results of the WR-EL method were compared to those of five individual CNN models. The results show that the identification accuracy increases by 32%, 19%, 15%, 11%, and 8%. Additionally, we proposed the SGDR-S algorithm, which improved the f1 scores of healthy wheat, stem rust wheat and leaf rust wheat by 2%, 3% and 2% compared to the SGDR algorithm, respectively. This method can more accurately identify wheat rust disease and can be implemented as a timely prevention and control measure, which can not only prevent economic losses caused by the disease, but also improve the yield and quality of wheat.

## 1. Introduction

Wheat is a main food crop, with a wide range of cultivation and many participating countries [1,2]. Both stem rust and leaf rust of wheat rely on spore transmission; they are spread rapidly and extensively, with moderate levels of wheat rust causing 20–30% yield reduction and severe levels causing more than 60% yield reduction or even crop failure [3,4,5]. Accurate and efficient identification of wheat rust can provide important guarantees for wheat disease control and yield improvement [6,7].

The urgent need for agriculture research on wheat disease detection has attracted attention, and many scholars have used a variety of methods to effectively identify wheat diseases from multiple perspectives [8,9]. For instance, Pujari et al. [10] used Radon transform and projection algorithms to process images of grain fungal diseases of wheat, maize, and sorghum, including leaf blight, leaf spot, powdery mildew, leaf rust, and smut, and used a support vector machine (SVM) classifier to identify and classify the features of these diseases. The method achieves a better classification effect, however the experimental sample of the study is negligible [11]. Johannes et al. [12] used mobile capture devices to obtain crop disease images and established an image recognition algorithm using statistical inference methods to solve the problem of identifying crop diseases under field conditions. Xu et al. [13] converted the RGB image of wheat leaf rust into a G single-channel grayscale image. The variance-SFFS method was used to screen the features, and then the set of screened features was identified using K-fold cross-validation (K set to 5) and SVM. This method can also select relevant features better and improve the accuracy of wheat powdery mildew and wheat rust identification. However, the variance-SFFS algorithm takes a longer time to screen features and requires further performance optimization [14]. Most of the above-mentioned traditional image processing and machine learning algorithms require image segmentation, feature extraction, etc. However, in practice, the spots are complex and irregular, which is not easily achieved by relying on extracting the best features from the image. Moreover, most of the studies focused only on the images of wheat leaf diseases, ignoring other parts such as stems and spikes, which lack practicality and completeness. Thus, the methods proposed by them were not convenient to detect wheat diseases in the field. There is, therefore, an urgent need to design a method for identifying wheat diseases with good robustness and high classification accuracy [15].

Owing to the development of deep learning techniques in the agricultural field, convolutional neural networks (CNNs) have been increasingly used for the recognition and classification of crop disease images, and CNNs have been proven to have better performance than traditional machine learning algorithms. Zhang et al. [16] constructed a five-layer structured neural network model for sample learning, and the learning process of the convolutional neural network (CNN) was controlled by the stochastic gradient descent (SGD) method. This model achieves the recognition of some common diseases, such as wheat blight and stripe rust, but the amount of sample data available for each disease is less, the controlled experiment are inadequate, and the whole study is not rigorous enough. Qiu et al. [17] processed the collected images of wheat spikes and established a deep convolutional neural network to train to identify the diseased areas in the images, which improves the efficiency of detecting wheat spike diseases in the field. The work of Johannes et al. in 2017 was improved by Picon et al. [18], who used an adaptive residual neural network for disease detection in wheat, further improving the identification of rust, Septoria (Septoria triciti), and Tan Spot (Drechslera triciti-repentis) in wheat. However, the number of images in different categories varied widely in the study dataset. Based on wheat disease features extracted by CNNs, feature fusion can obtain richer semantic information and improve the performance of the model [19,20]. In the above-mentioned studies, most of the wheat disease images used for training were insufficient and did not use any method to expand the samples. Moreover, the problem of large differences in the number of samples in different categories was not considered. In addition, in the case of choosing a model, accuracy was improved by increasing the depth of the network: this approach added too many parameters, in addition to making the model more complex [21]. Carefully, as the number of layers increases, the neural network becomes better fitted, but this also means that the functions represented are more complex and have more parameters, leading to longer training times and even overfitting. Although ensemble learning integrates multiple CNN models, not too many parameters are added by randomly selecting one CNN model for training in each iteration. The ensemble model has significantly fewer parameters compared to over-increasing the network depth. For example, Trung-Tin et al. [22] used an ensemble learning algorithm to classify and predict various nutrients that cause tomato diseases. The efficiency of ensemble learning was proved by comparing the prediction results of the single model and the integrated model. Prasath et al. [23] used integrated recurrent neural networks and CNNs for classification and clearly showed that the integrated model can avoid the drawbacks of individual classifier models and improve the accuracy of prediction. Currently, there are several articles that use the same dataset as we do, which was obtained from the ICLR Workshop and can be downloaded online from the Kaggle website. A more detailed description of this is given in Section 2. In the studies of GenaevS et al. [24] and Sood et al. [25] although better recognition results were achieved on this dataset using CNN models, they did not take into account the problem of the presence of erroneous images in the dataset, which obviously need to be removed.

At present, with the continuous development of CNNs for wheat disease identification, using only individual models, such as visual geometry group (VGG) [26] and Inception network [27], can no longer meet the current demands, because each model has its own work bias. Hence, ensemble learning can balance the advantages and disadvantages of various models, so that the classification task can be accomplished more excellently. To address the problem of the single model, this study proposed the use of ensemble learning to integrate several current mainstream classification frameworks, such as residual network (ResNet) [28] and densely connected networks (DenseNet) [29], and the bagging ensemble learning algorithm was used to extract the model in each training, which was the first time ensemble learning has been used for wheat rust identification. Moreover, the snapshot ensembling [30] method allowed a model that converged to multiple different local minima to be obtained in one training. A hyperparameter optimization search was used in this study, which could randomly select the data enhancement method, the iterative period of the learning rate in the SGDR-S [31] algorithm, etc., to reduce the interference caused by the artificial setting of abnormal values. In addition, the weighted cross-entropy (WCE) loss function was used to solve the problem of an imbalance in the number of samples.

## 2. Material

**Figure 1:**
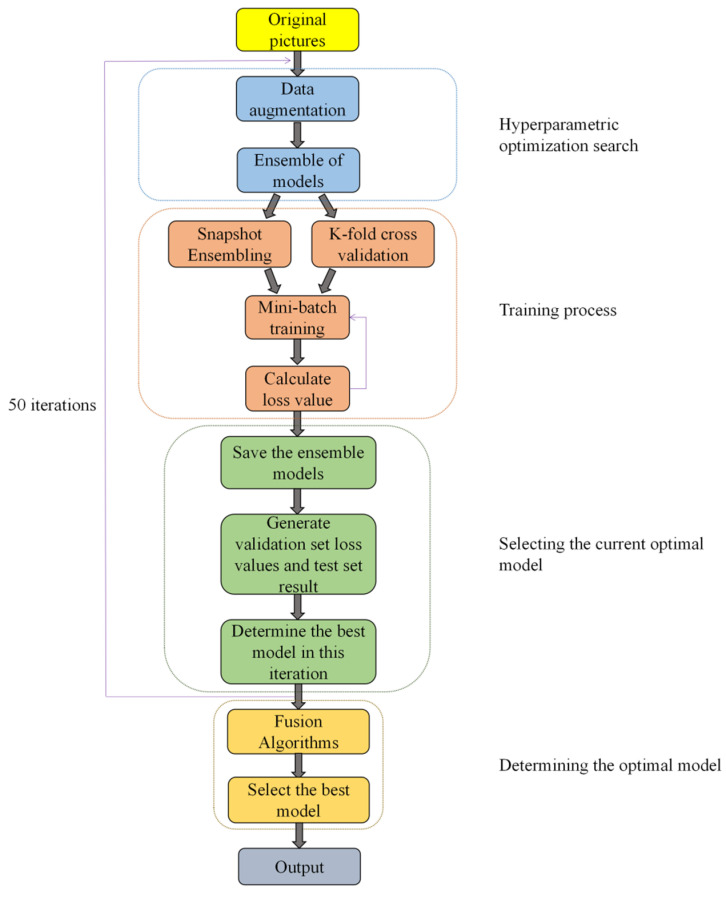
Workflow of this study.

Figure 1 illustrates the workflow of this study. The training set images of wheat rust were used as input, and the output was the probability that each image in the test set was classified into three categories (healthy wheat, stem rust wheat, and leaf rust wheat). First, data enhancement was performed on the training set images to expand the number of samples. Next, five network models, VGG 16, ResNet 101, ResNet 152, DenseNet 169, and DenseNet 201, were integrated, and the training process was performed by K-fold cross-validation and snapshot ensembling, which not only exploited the advantages of each network model but also improved the overall model generalization capability. Thereafter, the best model was selected in the current iteration process. Finally, the best model in the entire training process was selected and used in the testing process to obtain the final classification result.

**Figure 2:**
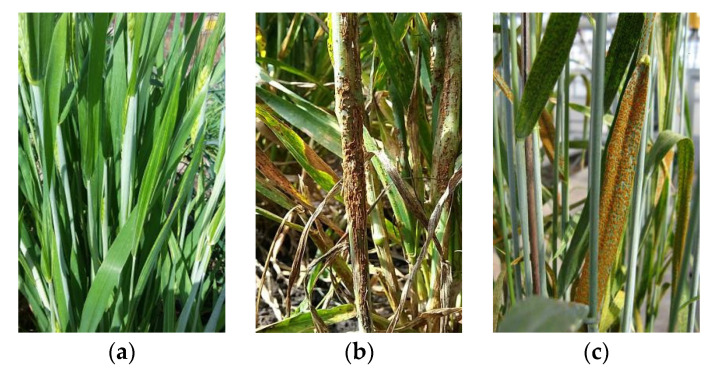
Sample images from the dataset used in this work. (**a**) Healthy wheat; (**b**) Stem rust wheat; (**c**) Leaf rust wheat.

Figure 2 is based on the dataset used in this research, which includes the three categories of healthy wheat, leaf rust wheat, and stem rust wheat, with image data from farm sites in Ethiopia and Tanzania, and from public images on Google Maps. The dataset can be obtain in the following link: https://zindi.africa/competitions/iclr-workshop-challenge-1-cgiar-computer-vision-for-crop-disease/data (accessed on 16 July 2022). Leaf rust occurs mainly on the leaf area, but it can also arise on the stem, and stem rust occurs mostly on the stem, but it may also appear on the leaf area. Therefore, it is important to judge not only the location of the disease but also the characteristics of the spot. In addition, some images contain both stem rust and leaf rust, but there is always a predominance of one rust over the other. In this case, the images were classified according to the type of wheat rust that was most prominent in the image.

In the dataset, there are 876 images in the training set, 610 images in the test set, and the original test set images have no labels. It is worth noting that among the 876 images in the training set, there are some images that appear repeatedly in the same category, and there are also some images that appear in different categories at the same time. We first remove these problematic images, and randomly divide 20% of the images in the training set into test data, and use the remaining 80% as training data. Therefore, there are 648 images in the training set and 162 images in the test set. Among them, there are 26, 66, and 70 pictures of healthy wheat, wheat with stem rust disease and wheat with leaf rust disease in the test set, respectively. The CNN model employed is pretrained on ImageNet and then finetuned on the above-mentioned dataset. The pre-training model and the model we use have the same convolutional layer in the feature extraction part, so training is not required at the beginning, and they should be frozen. Only train the last layer used for classification.

## 3. Methods

### 3.1. Data Augmentation

Even if placed in different orientations, the target can be classified robustly, which is considered to be the translation or rotation invariance of the network. More specifically, the CNN can be independent of the image direction, position, scale, luminance, or combinations between them, which is essentially a prerequisite for data enhancement. In addition, when using enhancement techniques, we must ensure that no extraneous data are added. There are many ways to enhance data, including rotation, random cropping and resizing, horizontal flipping, vertical flipping, and brightness enhancement. Some scholars have only used one or two of them in the entire training set. For example, Yu et al. [32] proposed BitMix, a data enhancement method for spatial image steganalysis, which only vertically rotates or horizontally flips the images of the small dataset used in image steganalysis. Hubert et al. [33] used CNNs for the grape image dataset for classification and detection tasks using only two ways of expanding the data, that is, mirroring along the x-axis and rotating by 15°. Nowadays, the use of more data enhancements is becoming more widespread. Pawara et al. [34] showed that data augmentation is very rich and that increasing the number of images in the training set using data augmentation techniques is useful for reducing overfitting and improving the overall performance of the CNN model. In addition, Sajjad et al. [35] experimentally evaluated various techniques for expanding datasets and the results proved that data augmentation technology is the most effective methods for improving performance. 

**Figure 3:**
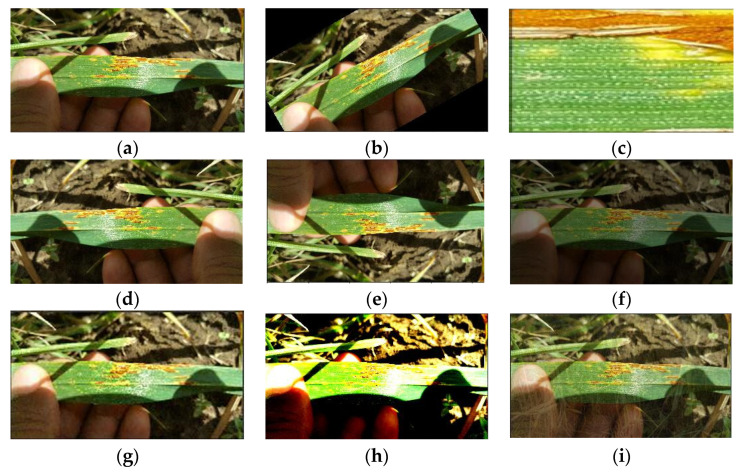
Comparison of the effects of 8 data enhancement methods. (**a**) Original image; (**b**) Rotated at any angle; (**c**) Randomly cropped and enlarged; (**d**) Horizontally flipped; (**e**) Vertically flipped; (**f**) Brightness enhanced; (**g**) Color dithered; (**h**) Contrast enhanced; (**i**) Mix-up enhancement.

Data augmentation has very positive effects on accuracy [36]. Therefore, eight data enhancement methods were collected in this study, and one of them was randomly selected for image processing using hyperparametric optimization search during each training process. This is conducive to improving the generalization capability of the model. The neural network model considers images to be different images after the data enhancement changes. An image of stem rust wheat is randomly selected from the dataset, and the image after eight different data enhancement methods is displayed, as shown in Figure 3.

### 3.2. Multiple Convolutional Neural Networks for Ensemble

CNNs are the cornerstone of deep learning for breakthrough results in computer vision [37,38], which consist of a series of filters, and the output is called feature maps. Each feature map is the output obtained by the convolution of a filter on the image, with the purpose of achieving the gradual abstraction of features from low-level to high-level. The formula of feature map is as follows:

(1) $$m=\frac{n-f+2p}{s}+1,$$

where m is the size of the feature map of the current layer, n is the size of the feature map of the previous layer, f is the size of the convolution kernel, p is the number of padding, and s is the step size.

**Figure 4:**
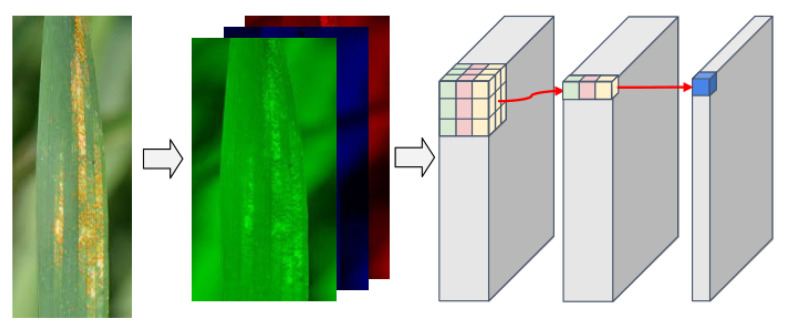
The information in the three channels of the RGB image enters the convolution layer, and after the convolution operation, the final feature map is obtained.

The CNN corrects the pixel-by-pixel migration and luminance effects by pooling operations, and extracts features with image migration and luminance invariance while preserving useful information in the image by continuously reducing the image resolution during the pooling process. Lin et al. [39] proposed that the 1 × 1 convolution kernel not only fuses the features of each channel, but also changes the size of the dimension and reduces the computational effort. Owing to the translation invariance of convolution, image-invariant feature extraction can be performed through a convolution operation. In practice, the information of the three channels of the RGB image is fed into the convolution layer, and then the information is convolved. Finally, the feature map corresponding to the channel size and the number of channels is generated, as shown in Figure 4.

**Figure 5:**
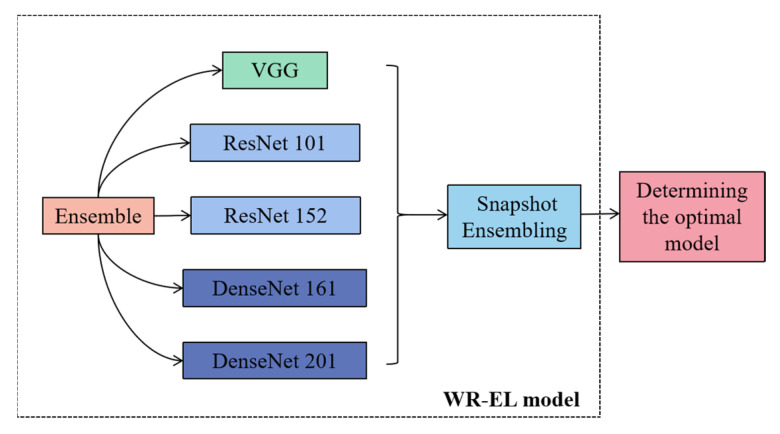
The WR-EL model structure.

The WR-EL model framework is shown in Figure 5, which integrates the five CNN models and selects the best model by using a snapshot ensemble algorithm in the model training. Different neural network models have different structural features and advantages, and the ensemble learning algorithm can integrate multiple neural network models. It can not only take into account the advantages of different models, but also ensure the correctness of the integration results. Even if the classifier produces the wrong results, it can also be solved through multiple training of the Bagging algorithm. In this study, VGG, ResNet, and DenseNet were used for feature extraction. The structures of these models are different. The ResNet model contains jump connections, the DenseNet model contains dense connections, and the VGG model does not contain these structures. This is the reason for their selection in this study. The specific introduction is as follows. The network structure of VGG enables the transfer of features through different layers. Each layer reads the feature information and passes it to the next layer. In this process, the features change, and at the same time, the information that needs to be saved will continue to be passed on. Each block in ResNet consists of a series of layers and a shortcut. The shortcut connects the input and output of the module together and adds at the element level, which is equivalent to crossing the middle layer and performing simple equivalent mapping. DenseNet has a denser connection. According to the forward propagation method, the feature maps of all previous layers are used as the input of the current layer, and the feature maps of the current layer are used as the input of all subsequent layers. A dense connection between each layer is achieved, and the information flow between each layer is maximized. This dense connection method is dimensional splicing, and it has rich feature information, and many feature maps can be obtained with less convolution. In addition, the densely connected network structure has a regularization effect, which can reduce the risk of overfitting. Currently, these models were also used in some of the latest studies. Abd Elaziz et al. [40] proposed a model for medical image classification based on integrated learning, integrating MobileNetV2 and DensNet 169 architectures as feature extraction backbone networks, which can perform the medical image classification task better. Hekal et al. [41] proposed an integrated deep learning model consisting of four CNNs that have undergone migration learning, namely AlexNet, ResNet-50, ResNet-101, and DensNet-201, and related studies on the accuracy and advantages of this integrated model.

In this study, VGG 16, ResNet 101, ResNet 152, DenseNet 161, and DenseNet 201 were used as the ensemble models, and the bagging ensemble learning algorithm was used to extract the network model by means of put back sampling. The optimal model generated in each iteration was saved, and the best model was selected using the subsequent fusion algorithm.

### 3.3. SGDR Algorithm

In general, local minima need to be avoided by all means, but Huang et al. [30] suggest that these local minima also contain useful information and can enhance the effect of the model. Therefore, the snapshot ensembling technology is used to make the network model cache a snapshot of the weights each time it converges to a local minimum, which enables the integration of multiple models without increasing the training cost.

The internal parameters of the model play a crucial role in training the model effectively and producing accurate results. Therefore, we use gradient descent algorithms to update and compute network parameters that affect the training of the model and the model output to approximate or reach the optimal values [42,43]. The gradient descent algorithm has two important control factors: the step size controlled by the learning rate and the direction specified by the gradient. The gradient descent algorithm was used to update the weights in a neural network model to minimize the loss function. The network updates the parameters as follows:

(2) $$x^{t+1}=x^{t}-\alpha \nabla \cdot J\left(x\right),$$

where α is the learning rate, $x^{t}$ denotes the current position, $x^{t+1}$ denotes the next position, $J\left(x\right)$ is the loss function with respect to x, and $\nabla \cdot J\left(x\right)$ denotes the gradient of this loss function. When the gradient is 0, the loss function is minimum. The speed of the weight update in the network model was determined by the learning rate α. In the stochastic gradient descent (SGD) algorithm, given an initial value of the learning rate, the learning rate is reduced as the number of training iterations increases, to bring the model infinitely close to the global minimum of the loss function [44]. However, during training, the algorithm tends to fall into local minima rather than global minima. The cosine annealing learning rate uses the cosine function to change the learning rate and can solve this problem better.

Loshchilov and Hutter [31] proposed the SGDR method, which is an SGD algorithm with restart, which can escape the local minima by suddenly increasing the learning rate and by finding the path to the global minimum. This method of combining the cosine annealing learning rate with restart uses the cosine function as a periodic function. The learning rate suddenly increases when each period reaches its maximum, and a new round of decay is restarted. Furthermore, Loshchilov and Hutter argue that it is better to increase the cosine period by multiplying each time it restarts. The iterative update formula for the cosine annealing learning rate is as follows:

(3) $$\alpha_{t}=\alpha_{\min}+\frac{1}{2}\left(\alpha_{\max}-\alpha_{\min}\right)\left[1+\cos\left(\frac{T_{cur}}{T}\pi \right)\right],$$

where $\alpha_{\min}$ and $\alpha_{\max}$ are intervals of variation in the learning rate, $T_{cur}$ is the number of iterations completed since the last restart to the current moment, and T specifies the number of iterations for the next restart. In our study, this was improved by proposing the SGDR-S algorithm, where the value of T was no longer a constant or multiplicatively increasing value but was determined by the product of the number of cycles of randomly selected training samples within a certain range for each training process. They can also be random values within a certain range. After several experiments, we set the number of cycles to 3–6 and trained 3–6 cycles at random in each cycle. This approach allowed to make more significant changes in the learning rate and greatly improved the training efficiency.

### 3.4. WCE Loss Function

In the classification task of wheat rust, the model’s output is the probability that the target belongs to each category, and the category with the highest probability is the one to which the target belongs. The softmax function can map each component of a vector to the interval [0,1] and normalize the output of the entire vector to ensure that the sum of all component outputs is 1 [45]. In this work, it was necessary to pass the extracted features through the softmax function to output the probability of each category, and then use the cross-entropy to calculate the loss value.

Cross-entropy can measure the difference between the true value and the predicted value. The smaller the value of cross-entropy, the better the model prediction. In addition, most datasets have the problem of category imbalance. When such problems occur, if the standard algorithm is used to calculate the loss, phenomena such as overfitting and underfitting will occur, and the final prediction results will also be greatly affected. Therefore, it is necessary to address the imbalance of data categories. In this study, we used the WCE loss function to address the problem of imbalance in the categories of the dataset. The formula for the WCE loss function is set as follows:

(4) $$L_{\mathrm{WCE}}=-\sum_{i=0}^{N-1}W_{i}y_{i}\log\left(p_{i}\right),$$

where N denotes the number of categories, $y=\left[y_{0},\cdot \cdot \cdot ,y_{N-1}\right]$ is the one-hot representation of the sample label, with $y_{i}=1$ when the sample belongs to category i and 0 for the rest, and $W=\left[w_{0},\cdot \cdot \cdot ,w_{N-1}\right]$ denotes the weight of the category. In this study, it was found that the probability of incorrect prediction for healthy wheat was very small, and the probability of incorrect prediction for leaf rust wheat was relatively high. Therefore, we set the cross-entropy weights for leaf rust and stem rust wheat to be relatively large during the training process. The final results show the superiority of the WCE loss function for the evaluation of the model in this dataset.

### 3.5. Fusion Algorithm

A total of 50 training sessions were performed in this study, and each training session produced an optimal model and corresponding loss value in the validation set and classification probabilities in the test set. Because k-fold cross-validation was used, the validation set loss contained the loss of all training images. The “replacement” idea was adopted to select the best verification set loss among the 50 evaluations, denoted as $V_{best}$, and save the corresponding test set classification probability, denoted as $P_{best}$. $V_{best}$ was used to multiply the verification set loss obtained from the remaining 49 evaluations and then square them to find a lower verification set loss. If there was a lower verification set loss, the same operation was performed on the probability of the corresponding test set. To select the best model in the integrated library, the algorithm can reduce overfitting and improve the generalization ability of the optimal model.

### 3.6. Performance Metrics

The confusion matrix is typically used in machine learning to evaluate or visualize the behavior of models in supervised classification contexts [46]. To make better judgments, some evaluation metrics based on confusion matrix are widely used. Accuracy is an overall measure of prediction accuracy, precision is a measure of how well all predicted positive results match the true state, recall represents the probability of a positive sample being predicted among the actual positive samples, and *F*1 score is the harmonic mean of precision and recall. The formulas for the prediction, recall, and *F*1 score show that all three metrics are concerned only with the positive category and ignore the performance of the negative category. To solve this problem, the predicted and true results can be viewed as two 0–1 distributions, and then the similarity of the two distributions can be measured by the Matthews’s coefficient of correlation (MCC). The value of MCC ranges from [−1, 1], where a value of 1 means that the prediction is exactly the same as the actual result, a value of 0 means that the prediction is not as good as the random prediction, and −1 means that the prediction is not at all consistent with the actual result. Thus, the MCC essentially describes the correlation coefficient between the predicted and actual results. The calculation formula is as follows.

(5) $$\mathrm{Accuracy}=\frac{\sum_{i=1}^{N}p_{ii}}{p},$$

(6) $$\mathrm{Precission}=\frac{1}{N}\overset{N}{\underset{i=1}{\sum }}\frac{p_{ii}}{p_{+i}},$$

(7) $$\mathrm{Recall}=\frac{1}{N}\overset{N}{\underset{i=1}{\sum }}\frac{p_{ii}}{p_{i+}},$$

(8) $$F1\;\mathrm{score}=\frac{1}{N}\overset{N}{\underset{i=1}{\sum }}\frac{2\times p_{ii}}{p_{+i}+p_{i+}},$$

(9) $$\mathrm{MCC}=\frac{1}{N}\overset{N}{\underset{i=1}{\sum }}\frac{p_{ii}\times \left(p-p_{+i}-p_{i+}+p_{ii}\right)-\left(p_{+i}-p_{ii}\right)\times \left(p_{i+}-p_{ii}\right)}{\sqrt[{2}]{p_{+i}\times p_{i+}\times \left(p-p_{+i}\right)\times \left(p-p_{i+}\right)}},$$

where $p_{ii}$ denotes the number of correctly predicted category i, p denotes the sum of all elements in the confusion matrix, $p_{i+}$ denotes the sum of row i in the confusion matrix, which is the number of categories in the actual situation, and $p_{+i}$ denotes the sum of column i in the confusion matrix, which is the number of categories in the predicted outcome. Here, $i=\left(1,\;2,\;\cdots ,\;N\right)$. In this paper, N is 3, which indicates three categories. 

## 4. Results and Analysis

Both the single and ensemble models used were pretrained with the ImageNet dataset, and the training process was performed using k-fold cross-validation with k set to 5. The experimental components of this study are discussed in the subsequent sections.

### 4.1. The Performance of the SGDR-S

**Figure 6:**
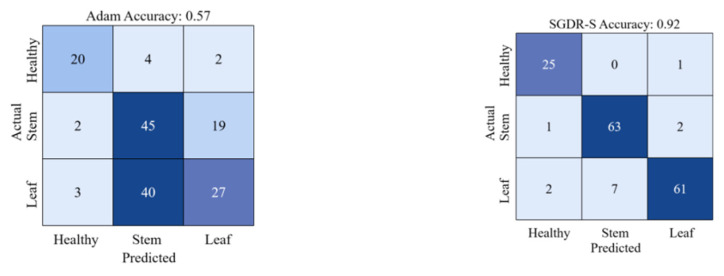
Classification accuracy and confusion matrix for both Adam and SGDR-S algorithms.

The purpose of gradient descent algorithms is to minimize the loss function, speed up the training process, and improve the accuracy of model training, and different gradient descent algorithms will achieve different results. Adam uses momentum and the adaptive learning rate to speed up convergence, which is one of the most commonly used gradient descent algorithms, and the SGDR uses the cosine annealing learning rate to speed up the decay of the learning rate. In this study, the number of iterations at the next restart in the SGDR was set to a random value within a certain range, as described in Section 3.3. Table 1 shows the classification results when using the Adam, the SGDR, and the SGDR-S algorithms, respectively. It can be seen that after using the SGDR-S algorithm, the model achieves the precision of 0.96 and 0.95 for healthy wheat and stem rust wheat, which is 4% and 3% higher than the precision of the SGDR algorithm, respectively. Although the precision of the SGDR-S algorithm for predicting leaf rust wheat was slightly lower at 0.87, it was also better than the SGDR algorithm. Furthermore, the F1 scores of all three categories improved significantly after using the SGDR-S algorithm. However, the Adam algorithm has low classification accuracy, recall and F1 scores for the three categories. Using the SGDR-S algorithm, the value of each category MCC is close to 1, and the average MCC value reaches 0.88, which is significantly higher than the average MCC value when using the SGDR algorithm. In particular, it can be seen from Figure 6 that the accuracy of Adam’s algorithm is only 0.57, while the accuracy of SGDR-S algorithm used in this study is 0.92.

### 4.2. Advantages of WCE Loss Function

**Figure 7:**
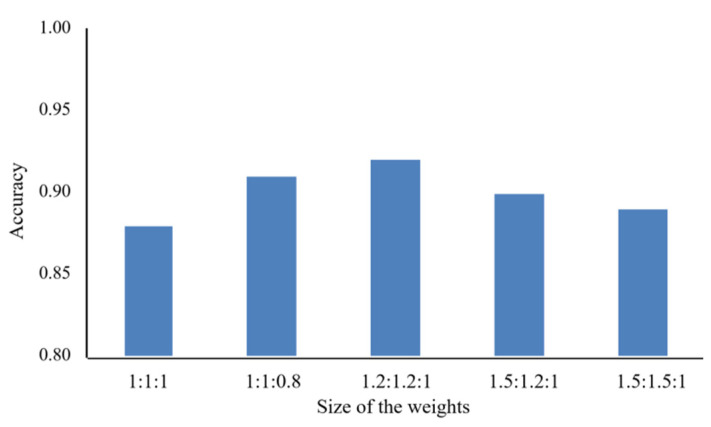
Accuracy of stem rust, leaf rust and healthy wheat at different weighting ratios.

The sample imbalance is expressed in terms of number and, in essence, in terms of learning difficulty. In the experiments, we found that all models had a small probability of predicting errors for healthy wheat, and a higher probability of predicting errors for both stem rust wheat and leaf rust wheat, as shown in Figure 7. The performance improved slightly after adding weights to the loss function, gradually increasing from 0.88 to 0.92. After several experiments, we finally chose a weighting factor of 1.2:1.2:1 for the three categories.

### 4.3. Performance Comparison of Individual Models and Wheat Rust Based on Ensemble Learning (WR-EL)

The ensemble model can integrate the learning ability of each model and improve the generalization ability of the final model. The advantage of VGG model is that all the convolutional kernels are replaced by 3 × 3 (1 × 1 is rarely used), which reduces the parameters and the computational effort, and makes the model have stronger discriminative ability. However, it is prone to a series of problems such as gradient disappearance and model degradation, which leads to difficult convergence and poor accuracy of the network. The advantage of the ResNet model is that it contains a residual structure, which can achieve constant and fast connectivity even without adding additional parameters, and can also solve the network degradation problem to a certain extent. Compared with it, the DenseNet model has more advantages. The DenseNet model is short-circuited by connecting features to achieve feature reuse, and the feature maps unique to each layer are relatively small. Moreover, the densely connected structure can greatly improve the back propagation of gradients, making the network easier to train. However, if not implemented properly, the DenseNet model consumes more GPU video memory during training. The WR-EL model is able to integrate the capabilities of multiple models and utilize the strengths of multiple CNN models to accomplish the classification task, resulting in better results than a single model. In addition, the WR-EL model has high classification accuracy and fast training speed, which can well enhance the expression ability of the model and reduce the classification loss. 

**Figure 8:**
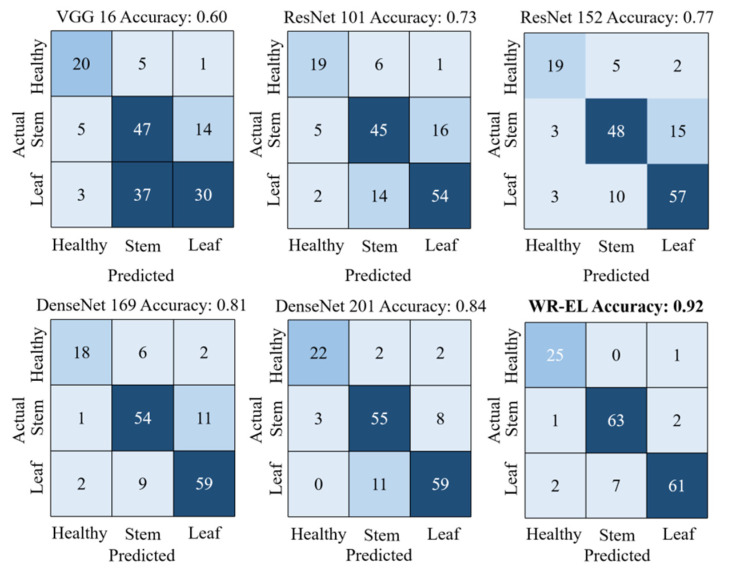
Confusion matrix for each model.

As can be seen from Table 2, the accuracy of several individual models on the test set ranged from 0.60–0.84. The WR-EL method performed better than the others, with an accuracy of 0.92 and a loss of less than 0.3. As shown in Figure 8, we showed the confusion matrix of each model. The specific classification of each model can be seen intuitively from the figure. Except for this research method, no other model can distinguish wheat from stem rust disease and wheat from leaf rust disease. In addition, in the task of identifying healthy wheat, this research method was also the best. It can be seen from the training time that this research method did not increase the training cost too much. It should be noted that the experiments in this study were run on workstations equipped with an NVIDIA GTX 1080 Ti graphics cards. In general, this showed that the classification effect of this research method was better, and it was desirable to use the ensemble model to detect wheat rust.

## 5. Discussion

### 5.1. Comparison of WR-EL and Single Model

In this article, a WR-EL method for wheat rust identification was presented. The approach used methods such as the integration of multiple CNNs and the snapshot ensemble algorithm to select the optimal model to apply it to the identification of wheat rusts. Different CNNs have different internal mechanisms and different abilities to extract complex image features. Dogan et al. [47] integrated a ResNet-based model to extract and fuse depth features to achieve high classification accuracy. Too et al. [48] used VGG 16, Inception V4, ResNet with 50, 101, and 152 layers, and DenseNet with 121 layers in their research of using leaf images to detect plant disease types for disease identification. Among them, the test accuracy rate of VGG was only more than 80%, while the test accuracy rates of other models were all above 90%. In the case of the same model architecture, the deeper the model, the higher the accuracy rate. This is also reflected in this research, the ResNet with 152 layers has a higher accuracy than the 101 layers, and the same is true under the DenseNet architecture. The same model was used for different tasks, and the results achieved were different. Most studies only investigate the recognition ability of a single model. However, it has been shown that the effect of integrating multiple CNNs is better than that of a single model in the research of facial expression classification based on integrated CNNs by Zhou et al. [49], which also proved this point of view. The effect of our WR-EL was much better than that of the single model. The WR-EL integrated the advantages of a variety of single CNNs and achieved an accuracy of 0.92, which shows that WR-EL outperforms the single model. In future work, we will consider validating the proposed method in this study on a dataset of different crops and different disease images and analyze its generalization ability. The WR-EL model will also be further improved to obtain better classification results.

### 5.2. The Superiority of SGDR-S Algorithm

Dede et al. [50] used the snapshot ensemble method to study a CNN model in the context of aerial scene classification. Their study demonstrated that the use of the snapshot ensemble method improved the classification results. The study used the snapshot ensembling method combined with the SGDR-S algorithm by taking snapshots at various local minima during training, and then fusing them together. It achieved the integration of a diverse and accurate model in one training process. This study improved the SGDR algorithm by changing the learning rate change cycle from a regular value (the cycle changes according to a fixed value or changes in multiple increments) to a random value within a certain range. This accelerated the speed of model training to a certain extent and improved the accuracy of the classification of wheat rust. The SGDR-S algorithm promoted classification accuracy from 0.90 to 0.92. In addition, the improved SGDR algorithm was compared to the Adam algorithm. Through experiments, we found that the classification accuracy of the Adam algorithm is only 0.57, which may be due to the adaptive learning rate algorithm may miss the maximum optimal solution [51]. For the superiority of SGDR-S algorithm, we will further apply it to more tasks to verify its performance.

### 5.3. Contribution of WCE Loss Function

In the case of imbalanced dataset categories, the network model will increase the possibility of misclassifications owing to different learning levels, and the WCE loss function can solve this problem more efficiently. The greater the weight coefficient, the greater the contribution. In the research done by Picon et al. [18], the sample size of the training set of wheat rust, Septoria, and Tan Spot was unbalanced. The sample size of wheat rust was 3338, the sample size of Septoria was 2744, and the number of Tan Spots was only 1568, which was less than half of the sample size of wheat rust, making the learning and training process of the deep CNN biased. When the final trained model was used for the classification of wheat rust, its accuracy was affected significantly, and the accuracy of the occurrence of wheat rust was higher than that of the other two. This study had considered this very carefully. The sample size of wheat stem rust and leaf rust was small, thus, the weight coefficient of the two increased, and the classification accuracy improved from 0.88 to 0.92 using the proposed method. Zhu et al. [52] effectively addressed the imbalance classification problem from an algorithmic perspective by designing a weighted SLFN model based on a multi-objective genetic algorithm. This study took into account unbalanced classification tasks by minimizing the average prediction accuracy and overall misclassification cost for both majority and minority classes. From the perspective of prediction accuracy, this algorithm also has certain advantages. In future work, we will consider over-sampling the categories with fewer samples during training or using an adaptive algorithm to determine the proportion of weights for different categories during training in order to better address the problem of category imbalance in the dataset.

## 6. Conclusions

In this paper, we proposed the WR-EL method that introduced an ensemble learning approach for wheat rust detection. This method integrated multiple convolutional neural networks without increasing the training difficulty and cost. In addition, this research proposes the SGDR-S algorithm to enhance the detection effect. To demonstrate the advantages of the WR-EL method, the recognition results of multiple individual models were compared with the proposed model, and the results showed that the WR-EL model had the best result with an accuracy of 0.92 and the lowest loss. To reflect the superiority of the SGDR-S algorithm, we compared it to the SGDR algorithm and the Adam algorithm. It was obvious from the results that the performance of the Adam algorithm was extremely poor, which may be due to the limitations of the adaptive learning rate in this algorithm. The Adam algorithm uses an exponentially shifted mean of the squared historical gradient to mitigate the overly rapid decay of the learning rate phenomenon, which limits each update to rely on gradient information from the last few iterations. Considering the imbalance of data categories, we tested different weighting factors for different categories and found that appropriately increasing the loss weights for both wheat stem rust and leaf rust categories could improve the final performance of the model, which is consistent with previous studies.

Future work will be conducted to validate the method proposed in this study based on a large dataset with different crops and different disease images, to analyze its generalization capability.

## Figures and Tables

**Table 1:** Performance of the Adam, SGDR, and SGDR-S algorithms.

Method	Class	Precision	Recall	F1 Score	MCC
Adam	Health	0.80	0.77	0.78	0.74
	Stem	0.51	0.68	0.58	0.22
	Leaf	0.56	0.39	0.46	0.17
SGDR	Health	0.92	0.88	0.90	0.87
	Stem	0.92	0.86	0.89	0.82
	Leaf	0.85	0.91	0.88	0.79
SGDR-S	Health	0.96	0.89	0.93	0.91
	Stem	0.95	0.90	0.93	0.87
	Leaf	0.87	0.95	0.91	0.85

**Table 2:** Performance of the WR-EL model.

Methods	Accuracy	Loss	Training Time	Params
VGG 16	0.60	2.29	547 min	138 M
ResNet 101	0.73	0.56	559 min	45 M
ResNet 152	0.77	0.49	575 min	60 M
DenseNet 169	0.81	0.45	570 min	14 M
DenseNet 201	0.84	0.32	595 min	20 M
WR-EL	0.92	0.29	589 min	14 M

## Data Availability

Not applicable.
